# Sexually dichromatic hybrids between two monochromatic duck species, the Chiloé wigeon and the Philippine duck

**DOI:** 10.1002/ece3.8253

**Published:** 2021-11-23

**Authors:** Jente Ottenburghs, Jan Harteman

**Affiliations:** ^1^ Wildlife Ecology and Conservation Wageningen University & Research Wageningen The Netherlands; ^2^ Forest Ecology and Forest Management Wageningen University & Research Wageningen The Netherlands; ^3^ Harteman Wildfowl Aviaries Winssen The Netherlands

**Keywords:** Anatidae, Anseriformes, hybrid fertility, hybridization

## Abstract

Captive bird hybrids can provide important data on certain traits, such as hybrid viability and fertility. In this paper, we describe four hybrids between the Chiloé wigeon (*Anas sibilatrix*) and the Philippine duck (*Anas luzonica*). These two species diverged about 13 million years ago and are found on different continents, making the occurrence of wild hybrids extremely unlikely. Hence, these captive hybrids provide a unique opportunity to learn more about the outcome of hybridization between these highly divergent species. One pair of hybrids mated and produced six unfertilized eggs, suggesting that hybrids between these species are infertile. Morphologically, the hybrids were slightly larger than the parental species, but had intermediate bill lengths. With regard to plumage patterns, the hybrids displayed characteristics of both parental species: Males developed the iridescent green head pattern of the Chiloé wigeon, whereas the females showed the dark crown and eye stripe of the Philippine duck. Interestingly, Chiloé wigeon and Philippine duck are both sexually monochromatic whereas the hybrids showed clear sexual dimorphism. These hybrids can thus lead to novel insights into the genetic and developmental basis of sexual mono‐ and dichromatism in ducks.

## Graphical abstract

In contrast to their parental species, captive hybrids between Chiloé wigeon and the Philippine duck show clear sexual dimorphism.

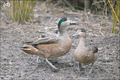

## 1 INTRODUCTION

Hybridization among captive birds is a relatively common phenomenon. According to the latest estimates, about 6% of documented bird hybrids are only known from captivity (Ottenburghs et al., 2015). Although the focus of hybridization research is mainly on wild hybrids, captive crosses can provide important information on particular traits that are difficult to measure in wild populations, such as viability and fertility of hybrid offspring (Arrieta et al., 2013; Lijtmaer et al., 2003; Tubaro & Lijtmaer, 2002). Many captive hybrids have been reported in the studbooks of zoos (Olney, 2003) or in the gray literature, such as magazines on bird breeding and husbandry. For example, in an article on putative mandarin duck (*Aix galericulata*) hybrids, Johnsgard (1968) referenced several articles from the *Avicultural Magazine* (Prestwich, 1960; Seth‐Smith, 1922). These primary sources are often hard to obtain and occasionally contain dubious information or anecdotal evidence, making it difficult to assess their reliability. It is thus important to clearly report on cases of captive hybridization and provide as many details as possible.

In this paper, we describe captive hybrids between the Chiloé wigeon (*Anas sibilatrix*) and the Philippine duck (*Anas luzonica*). The Chiloé wigeon is named after the Chiloé islands in southern Chile, although this species can be found as far north as Uruguay, Paraguay, and southern Brazil. The Philippine duck is restricted to the Philippines and nearby islands, such as Luzon, Masbate, Mindoro, and Mindanao. Within the Anatidae family, both species belong to different lineages that diverged about 13 million years ago (Gonzalez et al., 2009). The Chiloé wigeon is closely related to other wigeon species, such as the American wigeon (*Anas americana*) and the Eurasian wigeon (*Anas penelope*), whereas the Philippine duck can be found in a clade with the mallard (*Anas platyrhynchos*) and the American black duck (*Anas rubripes*), among others. To our knowledge, hybrids between Chiloé wigeon and Philippine duck have not been documented yet. Wild crosses are extremely unlikely given the distinct distributions of these species and no captive hybrids have been reported (McCarthy, 2006).

**FIGURE 1:**
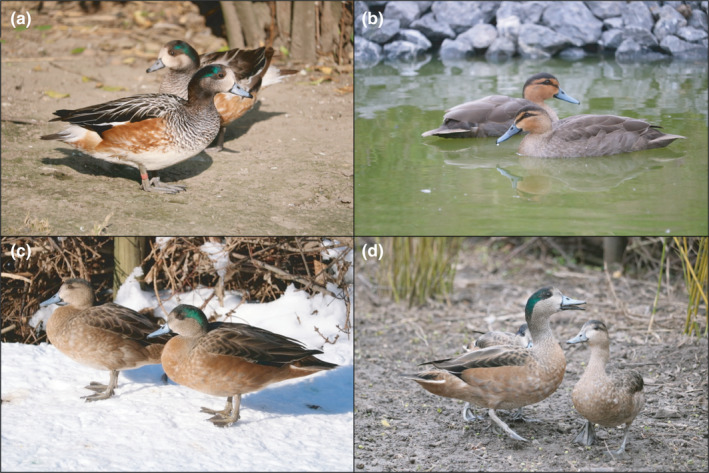
Pictures of the parental species—(a) Chiloé wigeon and (b) Philippine duck—and their hybrids (c–d). © Jan Harteman

Interestingly, Chiloé wigeon and Philippine duck are both sexually monochromatic (i.e., both sexes look alike). Male and female Chiloé wigeons show an iridescent green head pattern with a white forehead, although males tend to be somewhat brighter and glossier (Figure 1a, Todd, 1996). The sexes of the Philippine ducks also have similar plumage patterns with a bright rusty head that is marked by a dark crown (Figure 1b, Todd, 1996). Phylogenetic reconstructions indicate that the Chiloé wigeon and the Philippine duck have sexually dichromatic congeners in their lineages, suggesting that both species possess the genetic material to switch between plumage types (Lavretsky et al., 2014; Omland, 1997). Understanding the genetic and developmental basis of plumage patterns in the hybrids might thus provide some insights into the convergent evolution of sexual monochromatism in ducks.

In the following sections, we provide information on the morphology and fertility of captive hybrids between Chiloé wigeon and Philippine duck. In addition, we outline the current knowledge on the genetic and developmental basis of sexual mono‐ and dichromatism in ducks and speculate about possible mechanisms in Chiloé wigeon, Philippine duck, and their hybrids.

## 2 GENERAL DESCRIPTION OF THE HYBRIDS

The hybrids belong to the private waterfowl collection of Jan Harteman, where the parental species were housed together with canvasback (*Aythya valisineria*), white‐winged duck (*Asarcornis scutulata*), and Sunda teal (*Anas gibberifrons*). In the spring of 2020, a female Chiloé wigeon mated with a male Philippine duck and produced eight eggs. Six of these eggs were fertilized. The embryos in two of these eggs did not fully develop and were naturally aborted. The remaining four eggs hatched successfully on July 18, 2020, and the resulting hybrid offspring (two males and two females) were raised by hand. Later on, these hybrids formed two pairs, of which one pair produced a clutch of six unfertilized eggs. These observations suggest strong postzygotic isolation through hybrid breakdown.

### 2.1 Morphology

In contrast to the parental species, the hybrids showed clear sexual dimorphism. The males exhibited the iridescent green head pattern of the Chiloé wigeon, whereas the females developed the dark crown and eye stripe of the Philippine duck (Figure 1c–d). The body plumage of both sexes was rusty brown but lacked the characteristic patterning of the Chiloé wigeon. We measured the tarsus length and bill length of the four adult hybrids and compared it with data from other studies (Table 1). The hybrids had longer tarsi (on average 55.6 mm) than their parental species (*Anas luzonia*: 44–46 mm, *A*. *sibilatrix*: 40–43 mm). The larger size of the hybrids could be due to conditions in captivity where they have unlimited access to food, or they might show signs of hybrid vigor. However, the bill lengths of the hybrids were intermediate compared with the parental species (hybrids: on average 42.3 mm, *A*. *luzonia*: 46–52 mm, *A*. *sibilatrix*: 34–38 mm). Intermediate beak morphology of hybrids has been reported in other taxa, such as Darwin's Finches (Grant & Grant, 2016).

**TABLE 1:** Measurements of Chiloé wigeon (*Anas sibilatrix*), Philippine duck (*Anas luzonica*), and their hybrids

Specimen	Sex	Tarsus (mm)	Beak length (mm)	Source
*Anas sibilatrix*		40–43	34–38	Kear (2005)
*Anas luzonica*		44–46	46–52	Madge and Burn (1988)
Hybrid_1	Male	56.2	43.0	This study
Hybrid_2	Female	55.8	39.0	This study
Hybrid_3	Male	57.6	44.0	This study
Hybrid_4	Female	52.9	43.0	This study

### 2.2 Fertility

As mentioned above, one hybrid pair produced six unfertilized eggs, suggesting that hybrids between Chiloé wigeon and Philippine duck are infertile. Price and Bouvier (2002) calculated that it takes an average of 5 million years (for passerines) up to 17 million years (for non‐passerines) of evolution for species to produce infertile hybrids. The divergence time between Chiloé wigeon and Philippine Duck (ca. 13 million years) falls within this interval. However, it remains to be determined whether the male or the female hybrid (or both) are infertile. Most duck hybrids follow Haldane's Rule (Tubaro & Lijtmaer, 2002), which states that “when in the F1 offspring of two different animal races one sex is absent, rare, or sterile, that sex is always the heterozygous (i.e., heterogametic) sex.” In birds, females are the heterogametic sex (with ZW sex chromosomes), so it is likely that the female hybrid is sterile. However, the male hybrid may have produced deformed or immobile sperm cells (see, e.g., Alund et al., 2013). Finally, it is possible that fertilization was unsuccessful due to genetic incompatibilities between sperm and egg cells (Birkhead & Brillard, 2007). Unraveling the exact causes underlying the unfertilized eggs will thus require further investigation, for example, by quantifying misexpression of genes in reproductive tissues of the hybrids (Mugal et al., 2020).

## 3 THE GENETIC BASIS OF SEXUAL MONOCHROMATISM

Numerous bird species are sexually dichromatic, mostly with males showing more colorful and elaborate traits than females. The genetic and developmental mechanisms of sexual dichromatism are not fully understood yet and probably differ between species (Badyaev & Hill, 2003; Owens & Short, 1995). In ducks, plumage development seems to be determined by the amount of estrogen produced. The showy male plumage is the default state in both sexes, and the production of estrogen culminates in the development of cryptic female‐type plumage (Kimball & Ligon, 1999; Owens & Short, 1995). This mechanism has been confirmed experimentally in mallards (Haase & Schmedemann, 1992). The removal of gonads in both male and female ducks resulted in the maintenance of bright male plumage, while supplementation with estrogen during the molt leads to the development of the female‐like eclipse plumage. In addition, injecting testosterone and 5a‐dihydrotestosterone causes castrated male ducks to molt into eclipse plumage. This finding can be explained by the aromatization of androgens into estrogen. It is thus clear that sexual dichromatism in ducks is mainly estrogen‐dependent (Haase & Schmedemann, 1992; Kimball & Ligon, 1999).

Several duck species have independently evolved monochromatic sexes where males and females look alike (Omland, 1997). In some species, both sexes show cryptic female‐like plumage, while in other species, males and females exhibit bright, colorful plumage. These plumage patterns are probably the outcome of different estrogen levels: A repression of estrogen production will result in male‐like plumage, whereas an increase in estrogen production will lead to female‐like plumage. The production of these hormones might be controlled by particular modifier genes (Coyne et al., 2008; Williams & Carroll, 2009). Because sexual monochromatism arose independently in Chiloé wigeon and Philippine duck, it is likely that different modifier genes have evolved in both species, although these genes might be targeting similar hormonal pathways. The observation that male and female hybrids showed distinct plumage patterns suggests that these modifier genes might be located on the sex chromosomes. Genomic data and gene expression analyses (e.g., using RNAseq) could be applied to determine the location and identity of these modifier genes, highlighting the importance of captive bird hybrids in answering fundamental evolutionary questions.

## CONFLICT OF INTEREST

The authors declare that they have no conflict of interest.

## AUTHOR CONTRIBUTIONS

**Jente Ottenburghs:** Conceptualization (lead); Data curation (equal); Writing‐original draft (lead); Writing‐review & editing (equal). **Jan Harteman:** Conceptualization (supporting); Data curation (lead); Writing‐review & editing (equal).

## Data Availability

All the data accompanying this article are available in the main text.
